# Nationwide cohort study on the epidemiology and survival outcomes of thyroid cancer

**DOI:** 10.18632/oncotarget.19488

**Published:** 2017-07-22

**Authors:** Fu-Chao Liu, Huan-Tang Lin, Shu-Fu Lin, Chang-Fu Kuo, Ting-Ting Chung, Huang-Ping Yu

**Affiliations:** ^1^ Department of Anesthesiology, Chang Gung Memorial Hospital, Taoyuan, Taiwan; ^2^ College of Medicine, Chang Gung University, Taoyuan, Taiwan; ^3^ Department of Endocrinology, Chang Gung Memorial Hospital, Taoyuan, Taiwan; ^4^ Division of Rheumatology, Allergy and Immunology, Chang Gung Memorial Hospital, Taoyuan, Taiwan; ^5^ Division of Rheumatology, Orthopaedics and Dermatology, University of Nottingham, Nottingham, UK; ^6^ Office for Big Data Research, Chang Gung Memorial Hospital, Taoyuan, Taiwan

**Keywords:** thyroid cancer, epidemiology, survival outcome, National Health Insurance, Taiwan Cancer Registry

## Abstract

In the past three decades, the thyroid cancer incidence has surged globally. Herein, the Taiwan National Health Insurance database was used to identify thyroid cancer patients and to estimate the prevalence and incidence of thyroid cancer during 1997-2012. The Taiwan Cancer Registry and the National Death Registry databases were crosslinked to obtain information on the histological subtypes and survival rates. Joinpoint regression analysis was used for estimating the average annual percentage changes (APCs) in prevalence, incidence, and survival. The age-standardized incidence of thyroid cancer increased from 5.66 per 100,000 person-years in 1997 to 12.30 per 100,000 person-years in 2012, with an average APC of 5.1 (6.9 in males, 4.6 in females). Thyroid cancer was more prevalent in patients with high socioeconomic status and in urban areas. Papillary carcinoma was the most abundant subtype, with a 2.9-fold increase of incident cases noted during 1998-2012 (from 80.6% to 89.8% of all cases). Among the different treatments, partial thyroidectomy increased the most (average APC, 17.3). The overall survival rates by sex and subtype remained stable over time, with 5-year survival rates of 90.2% in 1997 and 92.4% in 2010. In conclusion, 2.2- and 4.2-fold increases in the incidence and prevalence of thyroid cancer, respectively, were observed during 1997-2012 in Taiwan. The surging incidence of thyroid cancer but stable survival rates, and mainly increased in the papillary subtype, altogether imply enhanced detection of subclinical lesions. A true increase due to environmental carcinogens might also be responsible, but warrant further investigations.

## INTRODUCTION

Thyroid cancer is the most common endocrine malignancy [1]. According to GLOBOCAN 2012, thyroid cancer accounted for 2.1% of the total cancer incidence in 2012, with a worldwide age-standardized incidence rate of 4.0 (1.9 in males, 6.1 in females) per 100,000 person-years and a worldwide mortality rate of 0.5 (0.3 in males, 0.6 in females) per 100,000 persons in 2012 [2]. While the incidences of most other common solid tumors in developed countries are either stable or decreasing, the incidence of thyroid cancer is surging in both sexes worldwide, largely coincident with the introduction of neck ultrasonography in the 1980s. Generally, patients with thyroid cancer have a favorable prognosis, with the 10-year survival rate estimated to be greater than 90% [1].

Thyroid cancer is a heterogeneous disease with distinct epidemiological and prognostic features for different histological subtypes. Thyroid follicular cells give rise to two major groups of thyroid cancers: differentiated (papillary and follicular) and undifferentiated (anaplastic, poorly differentiated) carcinomas [3]. Papillary and follicular thyroid carcinomas are more prevalent and have a relatively favorable prognosis, while the poorly-differentiated and anaplastic subtypes are much more rare and aggressive. Papillary thyroid carcinoma is the most abundant subtype, comprising 85-90% of all thyroid cancers. Most papillary thyroid carcinomas are clinically indolent, consistent with a simple genome and low mutational densities. Papillary thyroid microadenomas, defined as tumors <1cm in size, are the most commonly diagnosed thyroid cancers and are associated with long-term disease-free survival rates of >90% [4]. On the other hand, follicular thyroid carcinomas account for only 2-5% of thyroid cancers [3]. The follicular subtype is more aggressive than papillary carcinoma, mainly because it can metastasize via vascular invasion. Accordingly, it often presents with metastasis at the time of diagnosis.

Medullary thyroid carcinomas account for less than 5% of thyroid cancers, although they are responsible for approximately 13% of all thyroid cancer-related deaths [5]. Medullary thyroid carcinomas arise from neural crest-derived parafollicular C-cells, which do not accumulate radioiodine. These cells also secrete calcitonin and carcinoembryonic antigen, which are used as specific tumor markers. Most medullary thyroid carcinomas are sporadic, and usually develop in the fourth to sixth decade of life; however, up to 25% of cases are associated with a hereditary autosomal-dominant syndrome, known as multiple endocrine neoplasia type 2. Surgical excision is the primary curative therapy for patients with medullary thyroid carcinoma.

Anaplastic thyroid carcinomas account for approximately 1-2% of thyroid cancers and are the most aggressive subtype, associated with a mean overall survival of only 6 months, and more than 80% patients die within one year of diagnosis [3]. These highly invasive tumors should be surgically removed thoroughly and treated by adjuvant locoregional radiation therapy and chemotherapy.

Epidemiologic studies have reported increasing incidences of thyroid cancer worldwide in the last three decades, with improvements in the detection of thyroid cancer considered as the main reason underlying this rising trend [1]. The additional diagnoses from screening are mainly papillary thyroid carcinomas, which, as mentioned above, are a relatively prevalent histological finding with excellent prognosis in the general population and which are generally not considered a deadly disease [1, 6]. In fact, autopsy studies have revealed that at least one-third of adults without symptoms during their lifetime harbored small papillary thyroid carcinomas [1]. The other main explanation of the rising incidence of thyroid cancer is a true increase of thyroid carcinogenesis, driven by a higher exposure of potential risk factors such as radiation exposure, diabetes, excess weight, and/or environmental factors [1].

There are currently limited data regarding thyroid cancer epidemiology in Taiwan, and worldwide population-based cancer registries of the World Health Organization often have incomplete data of Taiwan. Previous reports from the Taiwan Cancer Registry database revealed that the age-standardized incidence of thyroid cancer increased from 1.44 per 100,000 person-years in 1980 to 13.3 per 100,000 person-years in 2012, as the second most increased solid cancer (in both sexes), with an average annual percentage change (APC) of 6.4 [7, 8]. However, information about the thyroid cancer subtypes and survival rates were not provided in that study. Additionally, estimating mortality rates based on the Taiwan National Health Insurance (NHI) database would result in overestimation of the true situations, because retraction from the NHI system does not always represent death. Therefore, we conducted this population-based epidemiologic study, mainly based on the NHI database and crosslinked with the Taiwan Cancer Registry and National Death Registry databases to improve the accuracy and reliability, with the aims to estimate the secular trends of the epidemiology and survival rates of thyroid cancer in Taiwan between 1997 and 2012.

## RESULTS

### Demographic data and geographic variations of patients with thyroid cancer

The eligible population in our study comprised of 21,952,273 registered NHI beneficiaries (49.33% male, 50.67% female) in 2012 in Taiwan. Of these, 25,711 patients (5,552 males and 20,160 females) with a thyroid cancer diagnosis during 1997-2012 were identified. The baseline characteristics of these thyroid cancer patients are presented in Table 1. Male thyroid cancer patients were diagnosed at an older mean age (49.83±15.80 years vs. 46.42±14.83 years) and had a significantly higher Charlson Comorbidity Index (CCI) score than female patients (2.66±2.95 vs. 1.90±2.51). The majority of thyroid cancer patients lived in urban areas (63.45%), while patients residing in rural areas comprised only 7.32% of cases. Furthermore, thyroid cancer patients more frequently had a professional occupation and high income level (quintiles 4 and 5, total 40.18%). These results of the socioeconomic status analysis indicated that thyroid cancer was more prevalent in high-income urban areas in Taiwan.

**Table 1:** Clinical characteristics of patients with thyroid cancer from 1997 to 2012

	Entire cohort (n=25,711)	By sex			By calendar year		
		Female (n=20,160)	Male (n=5,551)	*p*-Value	1997 (n=994)	2012 (n=2,656)	*p*-Value
**Age (yrs) (mean ± standard deviation)**	47.46±15.11	46.42±14.83	49.83±15.80	<.0001*	45.22±16.10	48.66±14.74	<.0001*
**Sex, No. (%)**							
Female	20,160 (78.41)	-- --	-- --	--	777 (82.31)	2,057 (77.45)	0.0017*
Male	5,551 (21.59)	-- --	-- --		167 (17.69)	559 (22.55)	
**Resident area, No. (%)**							
Urban	16,313 (63.45)	12,916 (64.07)	3,397 (61.20)	0.0006*	569 (60.28)	1,759 (66.23)	<.0001*
Suburban	7,177 (27.91)	5,521 (27.39)	1,656 (29.83)		273 (28.92)	710 (26.73)	
Rural	1,883 (7.32)	1,469 (7.29)	414 (7.46)	-	70 (7.42)	164 (6.17)	
Unknown	338 (1.31)	254 (1.26)	84 (1.51)		32 (3.39)	23 (0.87)	
**Income levels, No. (%)**							
Quintile 1	5,016 (19.51)	3,967 (19.68)	1,049 (18.90)	<.0001*	233 (24.68)	383 (14.42)	<.0001*
Quintile 2	5,583 (21.71)	4,651 (23.07)	932 (16.79)		346 (36.65)	240 (9.04)	
Quintile 3	4,605 (17.91)	3,762 (18.66)	843 (15.19)		66 (6.99)	737 (27.75)	
Quintile 4	5,033 (19.58)	4,028 (19.98)	1,005 (18.10)		143 (15.15)	592 (22.29)	
Quintile 5	5,296 (20.60)	3,631 (18.01)	1,665 (29.99)		143 (15.15)	694 (26.13)	
Unknown	178 (0.69)	121 (0.60)	57 (1.03)		13 (1.38)	10 (0.38)	
**Occupation, No. (%)**							
Dependents of the insured individuals	5,879 (22.87)	4,967 (24.64)	912 (16.43)	<.0001*	194 (20.55)	552 (20.78)	0.0047*
Civil servants, teachers, military personnel and veterans	1,258 (4.89)	859 (4.26)	399 (7.19)		55 (5.83)	129 (4.86)	
Non-manual workers and professionals	7,916 (30.79)	5,897 (29.25)	2,019 (36.37)		295 (31.25)	895 (33.70)	
Manual workers	8,629 (33.56)	7,001 (34.73)	1,628 (29.33)		347 (36.76)	852 (32.08)	
Other	2,029 (7.89)	1,436 (7.12)	593 (10.68)		53 (5.61)	228 (8.58)	
**Charlson index (mean ± standard deviation)**	2.07±2.63	1.90±2.51	2.66±2.95	<.0001*	1.68±2.32	0.71±1.88	<.0001*

* *p*<0.05.

Comparison based on calendar year between 1997 and 2012 revealed that the incident cases of thyroid cancer increased from 944 in 1997 to 2,656 in 2012, while the average CCI score decreased from 1.68±2.32 to 0.71±1.88. The female-to-male ratio of incident cases decreased from 4.65 in 1997 to 3.68 in 2012, whereas the average age of the thyroid cancer patients increased from 45.22±16.10 to 48.66±14.74 years, possibly due to the effect of an increased percentage of male cases (17.69% in 1997, 22.55% in 2012). The observed phenomenon of a higher prevalence in individuals with high socioeconomic status was even more pronounced when comparing the distribution in 2012 to that in 1997.

**Figure 1:**
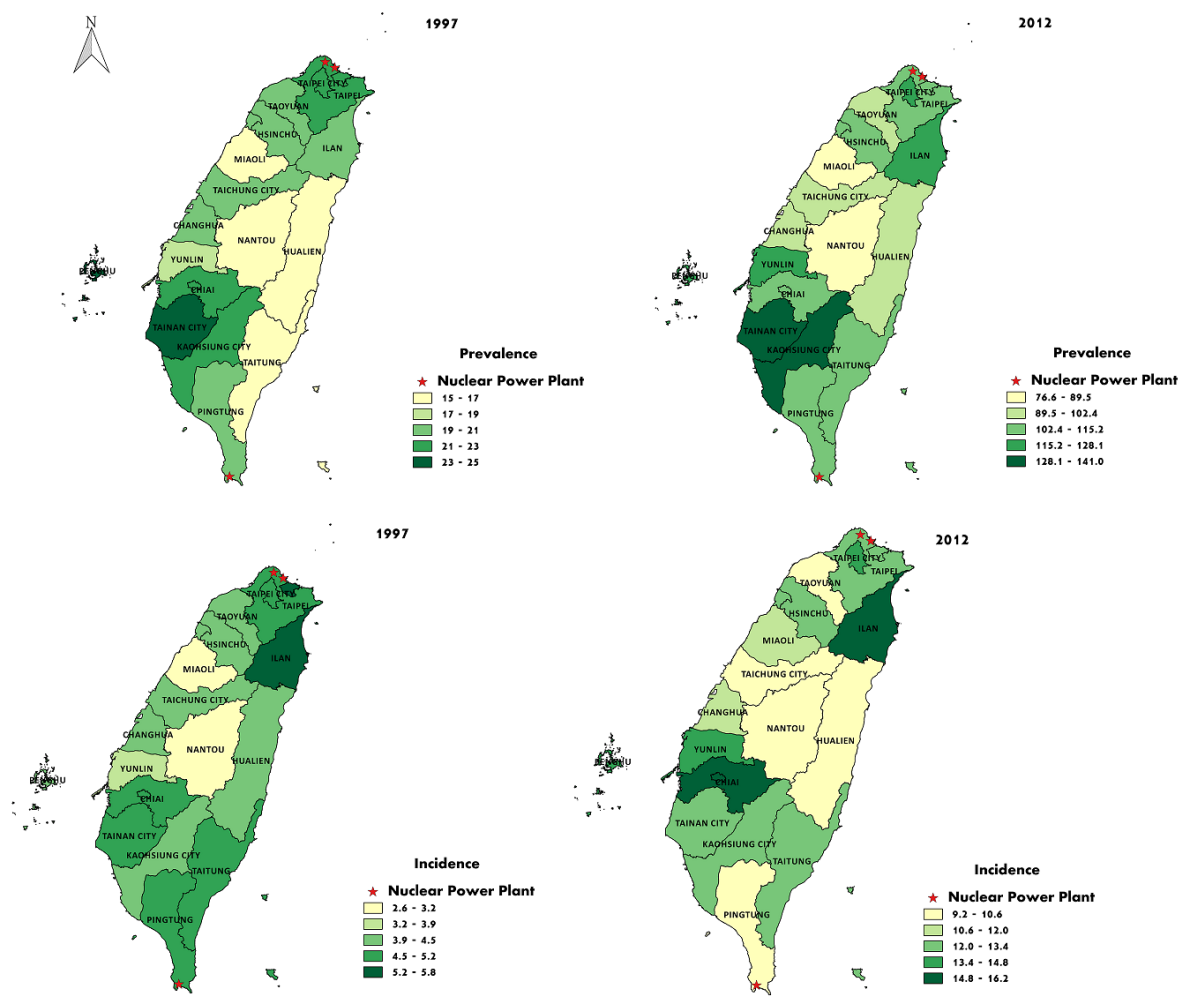
Geographic variations in the prevalence and incidence of thyroid cancer in Taiwan in 1997 and 2012, and their associations with the sites of nuclear power plants in Taiwan

The geographic variations in thyroid cancer prevalence and incidence in Taiwan are shown in Figure 1. The regions with higher prevalence were located in the urban and industrial areas, especially in Kaohsiung and Tainan, while the prevalence was relatively low in rural and mountainous areas. No obvious correlations between thyroid cancer prevalence and the sites of nuclear power plants in Taiwan was noted.

### Prevalence and incidence of thyroid cancer between 1997 and 2012

**Figure 2:**
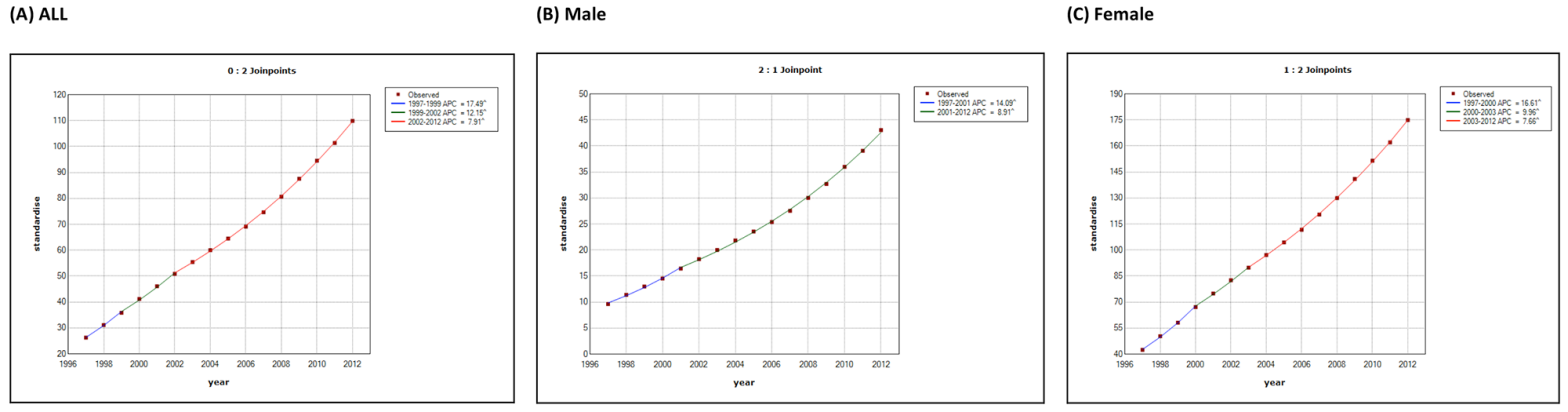

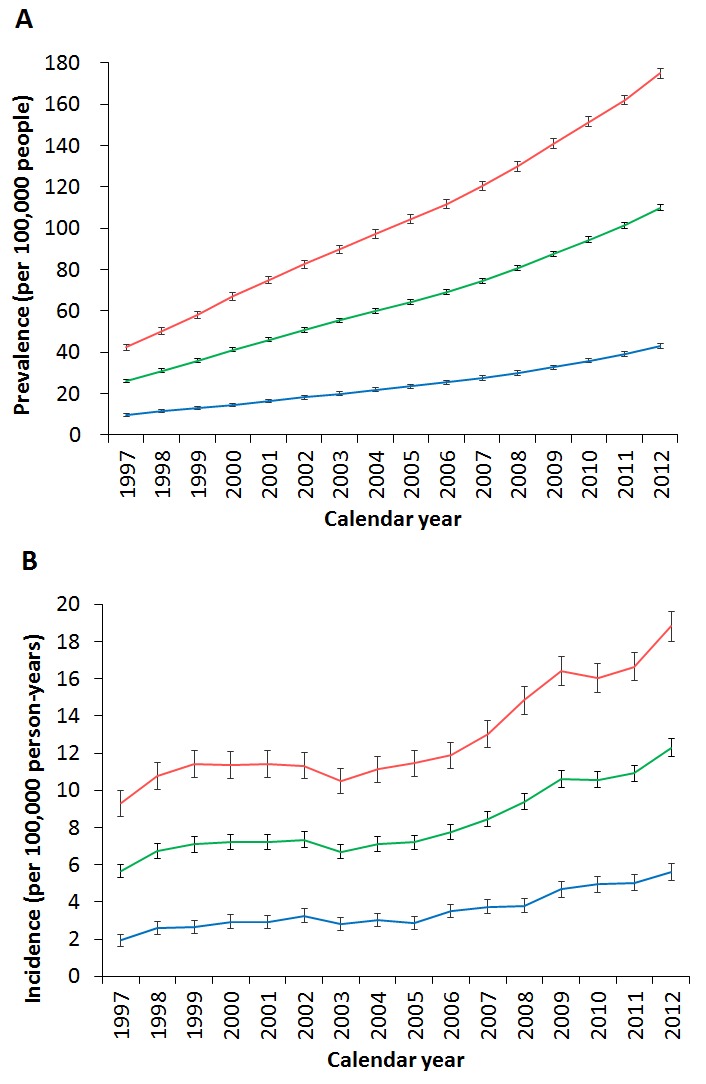
Differences by sex in the trends of **(A)** standardized prevalence and **(B)** standardized incidence of thyroid cancer in Taiwan between 1997 and 2012. (Red: female, green: total, blue: male.)

Tables 2 and 3 show the temporal trends in thyroid cancer prevalence and incidence in Taiwan during 1997-2012. The age-standardized prevalence of thyroid cancer was 26.2 (95% confidence interval: 25.43-27.03) per 100,000 persons in 1997 and 109.9 (108.49-111.26) per 100,000 persons in 2012. Joinpoint analysis of the thyroid cancer prevalence (Table 4) revealed that the average APC was 10.0 (9.5-10.5, p<0.05), with female patients [average APC, 10.3(9.8-10.7)] accumulating slightly faster than male cases [average APC, 9.9(9.4-10.3)]. On the other hand, the age-standardized incidence was 5.66 (5.29-6.04) per 100,000 person-years in 1997 and 12.30 (11.84-12.77) per 100,000 person-years in 2012, with the female-to-male ratio of age-standardized incidence decreasing from 4.8 in 1997 to 3.3 in 2012 (Figure 2). Joinpoint analysis of thyroid cancer incidence (Table 5) established 2 joinpoints, with an average APC of 5.1 (2.5-7.8, p<0.05). In detail, the average APCs of segments 1997-1999, 1999-2004, and 2004-2012 were 12.3 (-5.9 to 34.0), -1.1 (-5.9 to 4.1), and 7.4 (5.9 to 8.9)], respectively, with male patients showing a faster rise in incidence [average APC, 6.9(3.3-10.6)] than female cases [average APC, 4.6 (1.8-7.5)]. Overall, the standardized prevalence and incidence of thyroid cancer were 4.2-fold and 2.2-fold higher in 2012 than in 1997, respectively.

**Table 2:** Crude and standardized prevalence (per 100,000 persons) of thyroid cancer from 1997 to 2012

Year	Total			Male			Female		
	N	Crude	Standardized	N	Crude	Standardized	N	Crude	Standardized
1997	21,084,565	21.1 (20.46-21.70)	26.2 (25.43-27.03)	10,789,367	7.9 (7.38 -8.45)	9.6 (8.94-10.29)	10,295,198	34.9 (33.74-36.02)	42.4 (40.98-43.85)
1998	21,284,097	25.1 (24.43-25.78)	31.1 (30.22-31.94)	10,919,811	9.4 (8.87 -10.02)	11.4 (10.67-12.13)	10,364,286	41.6 (40.36-42.85)	50.2 (48.69-51.78)
1999	21,376,883	29.3 (28.55-30.00)	35.8 (34.92-36.76)	10,961,724	10.9 (10.25-11.48)	13.0 (12.19-13.73)	10,415,159	48.7 (47.32-50.00)	58.1 (56.47-59.76)
2000	21,437,626	33.9 (33.12-34.68)	41.1 (40.16-42.11)	10,964,786	12.2 (11.58-12.88)	14.5 (13.73-15.35)	10,472,840	56.6 (55.14-58.03)	67.0 (65.27-68.78)
2001	21,549,709	38.3 (37.49-39.14)	46.1 (45.04-47.09)	10,980,153	14.0 (13.29-14.69)	16.4 (15.56-17.26)	10,569,556	63.6 (62.07-65.11)	74.9 (73.09-76.77)
2002	21,490,211	42.7 (41.81-43.55)	50.8 (49.77-51.91)	10,911,148	15.7 (14.93-16.41)	18.2 (17.33-19.11)	10,579,063	70.5 (68.94-72.14)	82.6 (80.66-84.51)
2003	21,530,783	47.2 (46.30-48.13)	55.4 (54.28-56.49)	10,896,006	17.5 (16.69-18.26)	20.0 (19.08-20.93)	10,634,777	77.7 (76.01-79.36)	89.8 (87.84-91.81)
2004	21,692,825	52.1 (51.16-53.08)	60.0 (58.86-61.12)	10,942,756	19.4 (18.52-20.17)	21.8 (20.89-22.79)	10,750,069	85.5 (83.73-87.23)	97.1 (95.11-99.17)
2005	21,787,801	57.1 (56.04-58.05)	64.5 (63.34-65.65)	10,948,301	21.1 (20.26-21.98)	23.5 (22.55-24.51)	10,839,500	93.3 (91.52-95.15)	104.4 (102.3-106.5)
2006	21,879,926	62.2 (61.12-63.21)	69.1 (67.93-70.29)	10,958,204	23.2 (22.26-24.06)	25.4 (24.38-26.39)	10,921,722	101.3 (99.41-103.2)	111.7 (109.6-113.8)
2007	21,947,660	68.2 (67.12-69.30)	74.6 (73.40-75.82)	10,962,073	25.5 (24.58-26.47)	27.5 (26.49-28.56)	10,985,587	110.8 (108.8-112.8)	120.5 (118.3-122.6)
2008	22,009,377	74.9 (73.79-76.08)	80.6 (79.38-81.87)	10,962,089	28.2 (27.20-29.19)	30.0 (28.94-31.08)	11,047,288	121.3 (119.3-123.4)	129.9 (127.7-132.1)
2009	21,994,317	82.8 (81.64-84.05)	87.6 (86.29-88.85)	10,922,495	31.2 (30.18-32.28)	32.7 (31.59-33.80)	11,071,822	133.8 (131.6-135.9)	141.0 (138.7-143.3)
2010	22,009,193	91.2 (89.98-92.50)	94.5 (93.22-95.84)	10,900,482	34.9 (33.78-36.00)	36.0 (34.83-37.12)	11,108,711	146.5 (144.3-148.8)	151.5 (149.2-153.9)
2011	21,979,916	99.8 (98.43-101.1)	101.4 (100.03-102.72)	10,861,770	38.5 (37.35-39.69)	39.1 (37.88-40.25)	11,118,146	159.6 (157.2-161.9)	162.0 (159.7-164.4)
2012	21,952,273	109.9 (108.5-111.3)	109.9 (108.49-111.26)	10,828,933	43.0 (41.80-44.27)	43.0 (41.80-44.27)	11,123,340	175.0 (172.5-177.4)	175.0 (172.5-177.4)

**Table 3:** Crude and standardized incidence (per 100,000 person-years) of thyroid cancer from 1997 to 2012

Year	Total			Male			Female		
	Person-years	Crude	Standardized	Person-years	Crude	Standardized	Person-years	Crude	Standardized
1997	20,243,975	4.66 (4.37 -4.96)	5.66 (5.29-6.04)	10,270,668	1.63 (1.38 -1.87)	1.93 (1.63-2.24)	9,973,307	7.79 (7.24 -8.34)	9.30 (8.62-9.97)
1998	20,691,870	5.60 (5.28 -5.92)	6.76 (6.36-7.17)	10,572,162	2.28 (1.99 -2.57)	2.62 (2.28-2.97)	10,119,708	9.07 (8.48 -9.66)	10.79 (10.07-11.51)
1999	20,835,124	5.88 (5.55 -6.21)	7.09 (6.68-7.50)	10,666,163	2.17 (1.89 -2.45)	2.64 (2.28-2.99)	10,168,961	9.78 (9.18 -10.39)	11.42 (10.69-12.16)
2000	20,855,149	6.08 (5.75 -6.41)	7.20 (6.79-7.61)	10,667,910	2.48 (2.19 -2.78)	2.94 (2.57-3.31)	10,187,239	9.85 (9.24 -10.45)	11.35 (10.63-12.08)
2001	20,855,851	6.11 (5.78 -6.45)	7.23 (6.82-7.63)	10,651,291	2.52 (2.21 -2.82)	2.92 (2.55-3.28)	10,204,560	9.87 (9.26 -10.48)	11.42 (10.70-12.15)
2002	20,905,475	6.32 (5.98 -6.66)	7.35 (6.94-7.76)	10,647,131	2.84 (2.52 -3.16)	3.27 (2.89-3.65)	10,258,343	9.94 (9.33 -10.55)	11.33 (10.62-12.04)
2003	21,081,693	5.90 (5.57 -6.23)	6.69 (6.31-7.07)	10,695,909	2.47 (2.17 -2.77)	2.80 (2.45-3.15)	10,385,784	9.44 (8.85 -10.03)	10.49 (9.81-11.16)
2004	21,267,429	6.38 (6.04 -6.72)	7.12 (6.73-7.51)	10,752,053	2.73 (2.42 -3.05)	3.02 (2.67-3.37)	10,515,375	10.10 (9.49 -10.71)	11.12 (10.44-11.80)
2005	21,401,764	6.49 (6.15 -6.83)	7.21 (6.82-7.59)	10,782,313	2.60 (2.29 -2.90)	2.86 (2.51-3.20)	10,619,451	10.44 (9.83 -11.06)	11.45 (10.76-12.13)
2006	21,500,636	7.05 (6.69 -7.40)	7.75 (7.35-8.15)	10,790,603	3.23 (2.89 -3.56)	3.51 (3.14-3.89)	10,710,033	10.90 (10.27-11.52)	11.88 (11.18-12.57)
2007	21,586,501	7.83 (7.46 -8.20)	8.44 (8.03-8.85)	10,796,739	3.47 (3.12 -3.82)	3.73 (3.35-4.12)	10,789,762	12.19 (11.53-12.85)	13.03 (12.31-13.74)
2008	21,649,667	8.81 (8.42 -9.21)	9.38 (8.96-9.81)	10,797,325	3.60 (3.24 -3.96)	3.80 (3.42-4.18)	10,852,342	14.00 (13.29-14.70)	14.83 (14.07-15.58)
2009	21,670,116	10.14 (9.72 -10.57)	10.62 (10.18-11.07)	10,778,664	4.46 (4.06 -4.86)	4.69 (4.26-5.11)	10,891,452	15.76 (15.02-16.51)	16.41 (15.63-17.19)
2010	21,693,590	10.28 (9.85 -10.71)	10.58 (10.13-11.02)	10,757,963	4.81 (4.39 -5.22)	4.96 (4.53-5.39)	10,935,627	15.66 (14.92-16.41)	16.05 (15.28-16.81)
2011	21,638,725	10.77 (10.34-11.21)	10.92 (10.47-11.36)	10,700,141	4.95 (4.53 -5.37)	5.03 (4.60-5.46)	10,938,584	16.46 (15.70-17.23)	16.65 (15.88-17.42)
2012	21,586,050	12.30 (11.84-12.77)	12.30 (11.84-12.77)	10,651,206	5.62 (5.17 -6.07)	5.62 (5.17-6.07)	10,934,844	18.81 (18.00-19.62)	18.81 (18.00-19.62)

**Table 4:** Joinpoint analysis of thyroid cancer prevalence (per 100,000 persons) by sex in Taiwan, 1997-2012

	Thyroid cancer prevalence			Trend 1		Trend 2		Trend 3	
	1997	2012	Average APC	Years	APC (95%CI)	Years	APC (95%CI)	Years	APC (95%CI)
**Prevalence**									
**Total**	26.2 (25.4 to 27.0)	109.9 (108.5 to 111.3)	10.0 (9.5 to 10.5)*	1997-1999	17.5 (14.7 to 20.3)*	1999-2002	12.2 (10.1 to 14.3)*	2002 -2012	7.9 (7.8 to 8.0)*
**Male**	9.6 (8.9 to 10.3)	43.0 (41.8 to 44.3)	9.9 (9.4 to 10.3)*	1997-2000	16.6 (15.2 to 18.1)*	2000-2003	10.0 (7.8 to 12.1)*	2003- 2012	7.7 (7.5 to 7.8)*
**Female**	42.4 (41.0 to 43.9)	175.0 (172.5 to 177.4)	10.3 (9.8 to 10.7)*	1997-2001	14.1 (12.3 to 15.9)*	2001-2012	8.9 (8.7 to 9.1)*		

APC, annual percent change; CI, confidence interval. *P<0.05.

**Table 5:** Joinpoint analysis of thyroid cancer incidence (per 100,000 person-years) by sex in Taiwan, 1997-2012

	Thyroid cancer incidence			Trend 1		Trend 2		Trend 3	
	1997	2012	Average APC	Years	APC (95%CI)	Years	APC (95%CI)	Years	APC (95%CI)
**Incidence**									
**Total**	5.7 (5.3 to 6.0)	12.3 (11.8 to 12.8)	5.1 (2.5 to 7.8)*	1997-1999	12.3 (-5.9 to 34.0)	1999-2004	-1.1 (-5.9 to 4.1)	2004-2012	7.4 (5.9 to 8.9)*
**Male**	1.9 (1.6 to 2.2)	5.6 (5.2 to 6.1)	6.9 (3.3 to 10.6)*	1997-2000	12.4 (-2.7 to 29.8)	2000-2005	0.4 (-6.9 to 8.2)	2005-2012	9.4 (6.7 to 12.1)*
**Female**	9.3 (8.6 to 10.0)	18.8 (18.0 to 19.6)	4.6 (1.8 to 7.5)*	1997-1999	10.6 (-8.5 to 33.7)	1999-2004	-1.3 (-6.6 to 4.2)	2004-2012	7.1 (5.5 to 8.7)*

APC, annual percent change; CI, confidence interval. *P<0.05.

**Figure 3:**
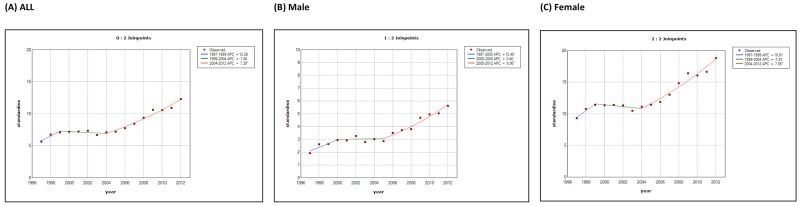

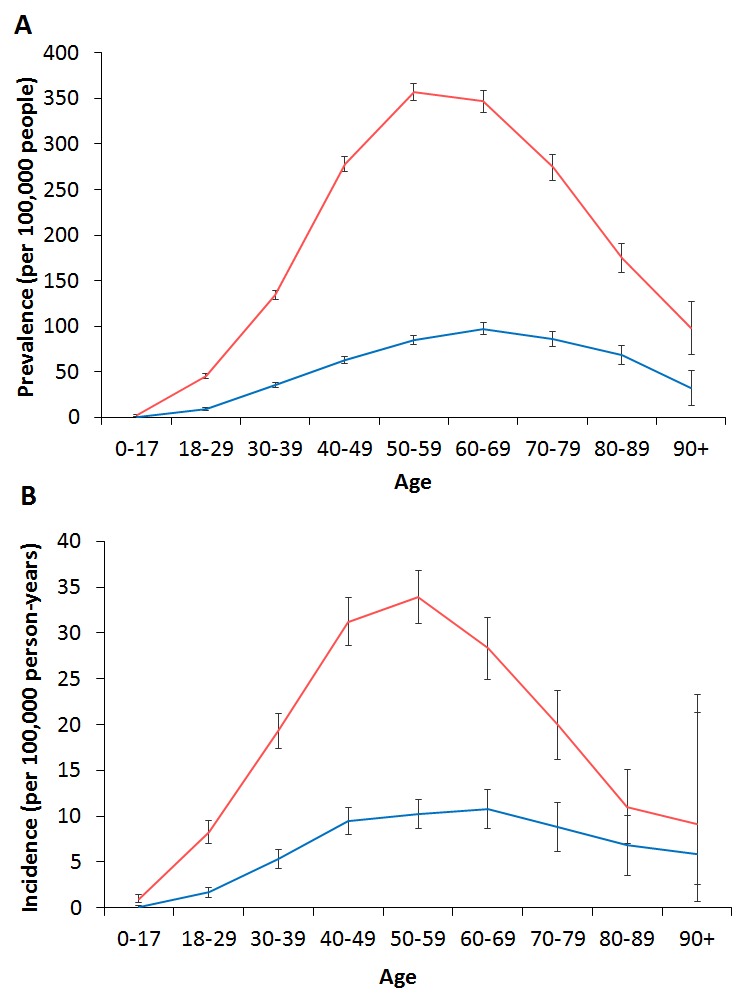
Differences by sex in the trends of **(A)** age-specific prevalence and **(B)** age-specific incidence of thyroid cancer in Taiwan between 1997 and 2012. (Red: female, blue: male.)

The age-specific prevalence and incidence of thyroid cancer are shown in Figure 3; the male thyroid cancer patients (peak incidence 60-69 years) were generally older than the female cases (peak incidence 50-59 years).

### Incidences and survival rates of different histological subtypes of thyroid cancer

We obtained information on the different thyroid cancer subtypes from the Taiwan Cancer Registry database. Table 6 shows the demographic data of patients according to their different subtypes. Patients with the papillary subtype had the youngest age at diagnosis (46.23±14.23 years), contained the highest proportion of females (79.11%), had the least comorbidities (CCI: 0.72±1.65), and the highest percentages of residence in urban areas, high income levels, and professional occupations. On the other hand, patients with the anaplastic subtype presented at the oldest age (70.58±11.74 years), had the most comorbidities (CCI: 2.07±2.76), and were the least likely to live in urban areas and to be classified as high socioeconomic status.

**Table 6:** Demographic data of patients with different thyroid cancer subtypes in Taiwan from 1997 to 2012

	Papillary thyroid carcinoma	Follicular thyroid carcinoma	Medullary thyroid carcinoma	Anaplastic thyroid carcinoma	Other type thyroid cancer
**Age (years) (mean ± standard deviation)**	46.23±14.23	48.87±18.22	51.56±14.61	70.58±11.74	58.63±19.62
**Sex, No. (%)**					
Female	18,374 (79.11)	1,615 (71.59)	233 (60.99)	218 (63.01)	806 (60.47)
Male	4,853 (20.89)	641 (28.41)	149 (39.01)	128 (36.99)	527 (39.53)
**Place of residence, No. (%)**					
Urban	13,894 (59.82)	1,254 (55.59)	215 (56.28)	144 (41.62)	648 (48.61)
Suburban	6,218 (26.77)	685 (30.36)	111 (29.06)	90 (26.01)	382 (28.66)
Rural	1,542 (6.64)	189 (8.38)	34 (8.90)	37 (10.69)	165 (12.38)
Unknown	1,573 (6.77)	128 (5.67)	22 (5.76)	75 (21.68)	138 (10.35)
**Income levels, No. (%)**					
Quintile 1	4,689 (20.19)	476 (21.10)	83 (21.73)	75 (21.68)	334 (25.06)
Quintile 2	3,990 (17.18)	488 (21.63)	75 (19.63)	74 (21.39)	352 (26.41)
Quintile 3	4,794 (20.64)	448 (19.86)	65 (17.02)	59 (17.05)	189 (14.18)
Quintile 4	4,601 (19.81)	410 (18.17)	82 (21.47)	37 (10.69)	169 (12.68)
Quintile 5	5,059 (21.78)	398 (17.64)	72 (18.85)	45 (13.01)	186 (13.95)
Unknown	94 (0.40)	36 (1.60)	5 (1.31)	56 (16.18)	103 (7.73)
**Occupation, No. (%)**					
Dependents of the insured individuals	5,100 (21.96)	661 (29.3)	81 (21.2)	117 (33.82)	392 (29.41)
Civil servants, teachers, military personnel and veterans	1,087 (4.68)	97 (4.30)	23 (6.02)	5 (1.45)	27 (2.03)
Non-manual workers and professionals	7,381 (31.78)	558 (24.73)	103 (26.96)	18 (5.20)	150 (11.25)
Manual workers	7,423 (31.96)	685 (30.36)	122 (31.94)	109 (31.50)	482 (36.16)
Other	2,236 (9.63)	255 (11.3)	53 (13.87)	97 (28.03)	282 (21.16)
**Charlson index (mean ± standard deviation)**	0.72±1.65	0.84±1.88	1.05±2.13	2.07±2.76	1.74±2.55

**Figure 4:**
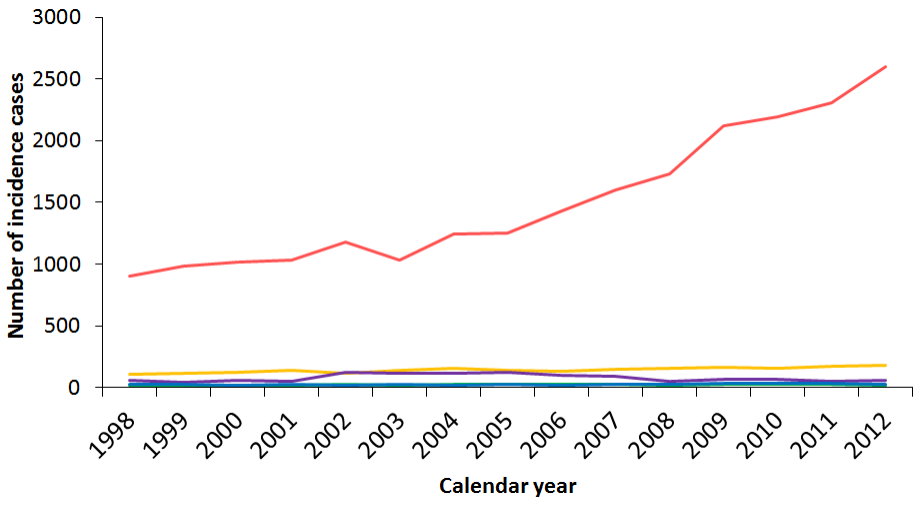
Secular trends of the incidence cases of different thyroid cancer subtypes in Taiwan between 1998 and 2012 (Red: papillary thyroid carcinoma, yellow: follicular thyroid carcinoma, blue: medullary thyroid carcinoma, green: anaplastic thyroid carcinoma, purple: other types.)

**Figure 5:**
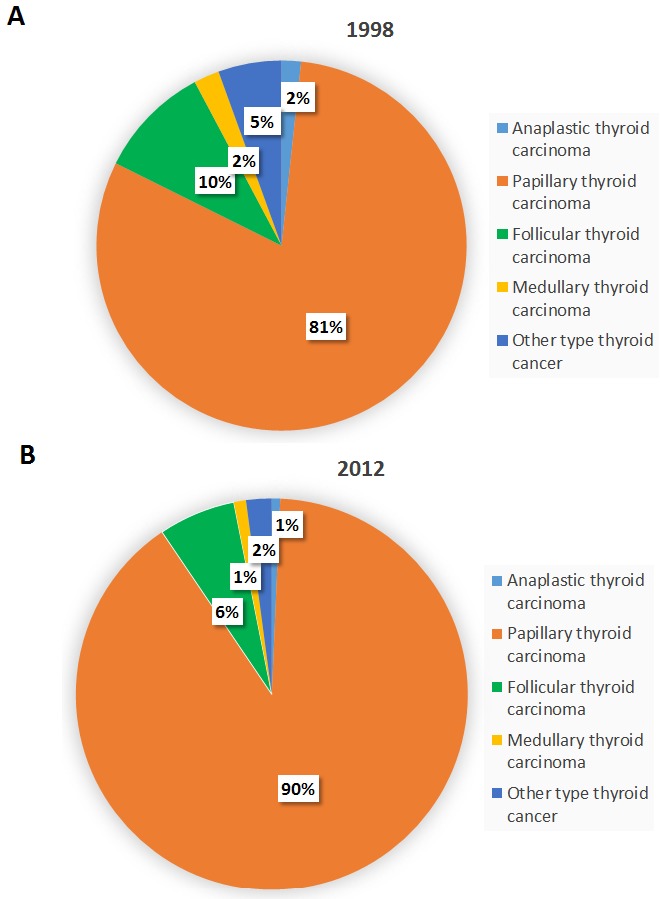
The percentages of different thyroid cancer subtypes in Taiwan in **(A)** 1998 and **(B)** 2012.

Figure 4 illustrates the incidence trends of different thyroid cancer subtypes in Taiwan between 1998 and 2012. Papillary thyroid carcinoma was the most abundant subtype with the most rapid surge in incidence, growing from 80.6% of the total cases in 1998 (904 incident cases) to 89.8% in 2012 (2,601 incident cases) (Figure 5). The percentage of the other histological subtypes gradually decreased from 1998 to 2012 (follicular subtype 9.9% to 6.4%, medullary subtype 2.2% to 1%, anaplastic subtype 1.7% to 0.7%, and other subtypes 5.5% to 2.1%) despite the actual numbers of incident cases of the follicular subtypes increasing 1.6-fold during this period. The papillary-to-follicular incidence ratio grew from 8.14 in 1998 to 14.13 in 2012.

Although the incident percentages of the different subtypes changed significantly during this period, their survival rates were essentially stable. Joinpoint analysis of the survival rates for different subtypes (Table 7) showed no statistically significant changes during 1997-2012, with 5-year survival rates in 2010 of 95.5%, 89.3%, 80.6%, 36.4%, and 7.4% for the papillary subtype, follicular subtype, medullary subtype, other subtypes, and anaplastic subtype, respectively. Owing to the accelerating incidence rate and more favorable survival, the papillary subtype has become increasingly predominant over time.

**Table 7:** Joinpoint analysis of overall survival by different thyroid cancer subtypes in Taiwan, 1998-2012

Thyroid cancer subtypes	Overall survival rate (%)		Average APC
	1998	Last year^a^	
**Papillary thyroid carcinoma**			
1 year survival rate	98.2% (96.92-98.91)	98.7% (98.17-99.06)	0.02 (-0.01 to 0.05)
2 year survival rate	97.2% (95.81-98.20)	98.0% (97.42-98.50)	0.03 (-0.01 to 0.07)
5 year survival rate	94.5% (92.62-95.90)	95.5% (94.49-96.36)	0.05 (-0.04 to 0.15)
**Follicular thyroid carcinoma**			
1 year survival rate	95.3% (89.04-98.01)	97.3% (93.56-98.85)	0.05 (-0.12 to 0.22)
2 year survival rate	93.4% (86.65-96.80)	95.1% (90.75-97.41)	0.19 (-0.07 to 0.45)
5 year survival rate	86.8% (78.72-91.96)	89.3% (81.36-93.99)	0.24 (-0.35 to 0.84)
**Medullary thyroid carcinoma**			
1 year survival rate	92.9% (59.08-98.96)	89.7% (71.26-96.54)	-0.30 (-0.84 to 0.23)
2 year survival rate	92.9% (59.08-98.96)	89.7% (71.26-96.54)	-0.72 (-1.27 to -0.17)*
5 year survival rate	78.6% (47.25-92.54)	80.6% (61.91-90.80)	-0.20 (-1.07 to 0.68)
**Anaplastic thyroid carcinoma**			
1 year survival rate	29.4% (10.71-51.15)	13.8% (4.35-28.61)	-5.51 (-10.08 to -0.72)*
2 year survival rate	23.5% (7.31-44.92)	6.90% (1.22-19.75)	-7.45 (-11.59 to -3.13)*
5 year survival rate	17.6% (4.35-38.30)	7.40% (1.30-21.03)	-17.34 (-54.97 to -0.68)
**Other type thyroid cancer**			
1 year survival rate	71.2% (57.81-80.99)	63.3% (49.85-74.11)	-1.34 (-3.15 to 0.51)
2 year survival rate	69.5% (56.04-79.55)	53.3% (40.01-64.96)	-1.98 (-4.28 to 0.37)
5 year survival rate	64.4% (50.81-75.14)	36.4% (23.84-49.00)	-3.08 (-6.53 to 0.49)

APC, annual percent change; *P<0.05.

^a^ The last year for the 1-year and 2-year survival rate is 2012; the last year for the 5-year survival rate is 2010.

### Treatments for thyroid cancer

**Figure 6:**
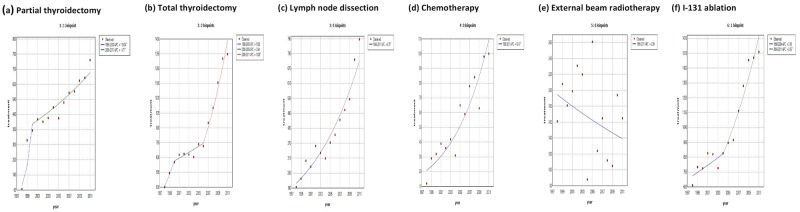

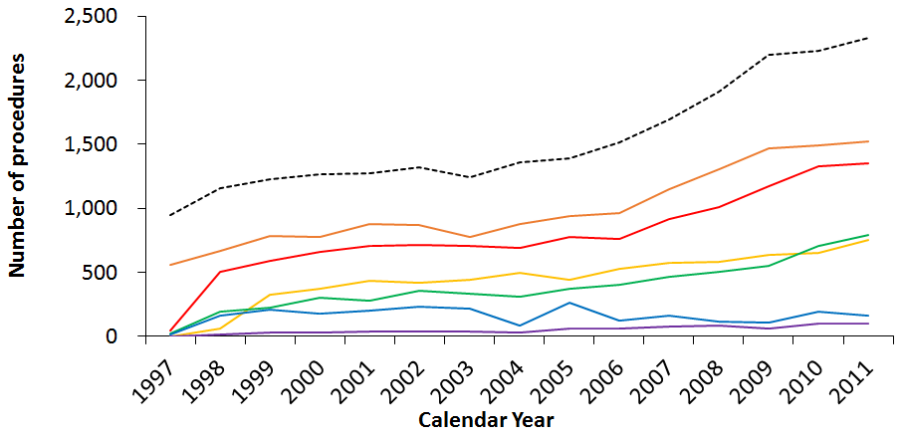
Secular trends of the numbers of different treatment procedures for thyroid cancer in Taiwan between 1997 and 2011 (Dotted line: the incidence cases at that specific year, orange: I-131 ablation, red: total thyroidectomy, yellow: partial thyroidectomy, green: lymph node dissection, blue: external beam radiotherapy, purple: chemotherapy.)

We calculated and compared the numbers of different treatment procedures performed after the diagnosis of thyroid cancer. Among the entire cohort in Taiwan during 1998-2011, 86% of thyroid cancer patients in Taiwan received partial or total thyroidectomy, and 61% received radioiodine ablation, while 15% of patients received no treatment at all after diagnosis (Table 8). Analysis of the thyroid cancer treatments by sex revealed that male thyroid cancer patients received more advanced treatments than female cases, including neck dissection, chemotherapy, and external beam radiotherapy. This result might be due to male patients having a worse prognosis than females, thus requiring more adjuvant therapies. Joinpoint analysis of the treatment procedures showed generally rising trends during 1998-2011, owing to the increase in incident cases, except for external beam radiotherapy [average APC, -2.54 (-6.91 to 2.04)] (Table 9). The most sharply accelerating trend was noted for the partial thyroidectomy, which showed an 11.5-fold increase from 1998 to 2011, with an average APC of 17.3 (14.2 to 20.5, p<0.05). Comparing partial to total thyroidectomy, partial thyroidectomy accounted for 35.6% of thyroidectomies in 2011 but only 11.5% in 1998. The average APCs of major treatments such as total thyroidectomy [8.39 (5.93 to 10.9), p<0.05] and I-131 ablation [6.32 (4.09 to 8.59), p<0.05] even outpaced the average APC of thyroid cancer incidence during the period. Figure 6 shows the secular trends of different treatment procedures of thyroid cancer in Taiwan from 1997 to 2011.

**Table 8:** The numbers of different treatment procedures for patients with thyroid cancer by sex in Taiwan from 1997 to 2011

Treatments^a^	Entire cohort (n=25711)	By sex				
		Female(n=20160)	% in female pts	Male(n=5551)	% in male pts	*p*-Value
**Partial thyroidectomy**	8,573	6,832	33.89%	1,741	31.36%	0.0004*
**Total thyroidectomy**	13,649	10,708	53.12%	2,941	52.98%	0.8598
**Lymph node dissection**	6,972	5,237	25.98%	1,735	31.26%	<0.0001*
**Chemotherapy**	1,030	676	3.35%	354	6.37%	<0.0001*
**External beam radiotherapy**	2,600	1,906	9.45%	694	12.5%	<0.0001*
**I-131 ablation**	15,718	12,305	61.04%	3,413	61.48%	0.5445

^a^ All treatments for thyroid cancer occurred after the diagnosis of thyroid cancer. *P<0.05.

**Table 9:** Joinpoint analysis of the number of treatment procedures for thyroid cancer in Taiwan, 1998-2011

	Number of treatments			Trend 1		Trend 2		Trend 3	
	1998	2011	Average APC	Years	APC (95%CI)	Years	APC (95%CI)	Years	APC (95%CI)
**Partial thyroidectomy**	65	749	17.3 (14.2 to 20.5)*	1998-2000	119.3 (81.5 to 165.1)*	2000-2011	4.71 (3.38 to 6.06)*		
**Total thyroidectomy**	502	1353	8.39 (5.93 to 10.9)*	1998-2000	15.0 (-0.05 to 32.3)	2000-2006	2.64 (-0.53 to 5.91)	2006-2011	13.0 (9.51 to 16.6)*
**Lymph node dissection**	194	788	9.75 (8.07 to 11.5)*	1998-2011	9.75 (8.07 to 11.5)*				
**Chemotherapy**	12	100	13.6 (9.51 to 17.9)*	1998-2011	13.6 (9.51 to 17.9)*				
**External beam radiotherapy**	161	165	-2.54 (-6.91 to 2.04)	1998-2011	-2.54 (-6.91 to 2.04)				
**I-131 ablation**	670	1524	6.32 (4.09 to 8.59)*	1998-2004	2.99 (-0.94 to 7.07)	2004-2011	9.26 (5.95 to 12.7)*		

APC, annual percent change; CI, confidence interval. *P<0.05.

### Analysis of thyroid cancer survival rates by sex and Charlson Comorbidity Index

**Figure 7:**
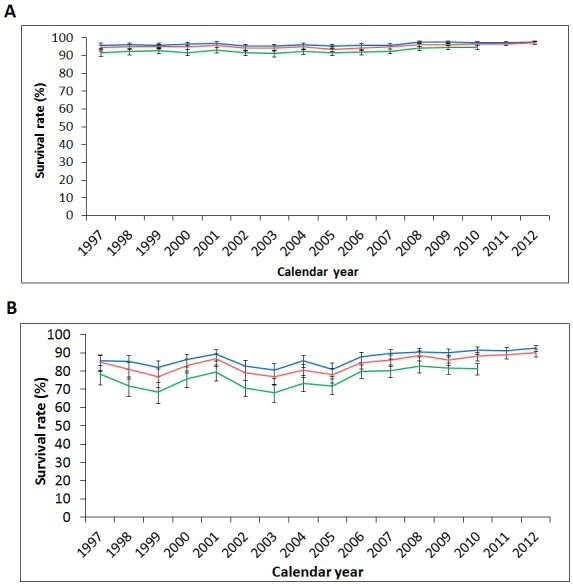
Secular trends of the one-, two-, and five-year survival rates of thyroid cancer in **(A)** female and **(B)** male patients in Taiwan from 1997 to 2012. (Blue: 1-year survival rate, red: 2-year survival rate, green: 5-year survival rate.)

The 1-, 2-, and 5-year thyroid cancer survival rates in Taiwan were generally stable or mildly improved (Figure 7), with slightly increased average APCs during the study period (Table 10). The latest calculated 1-, 2-, and 5-year survival rates were as follows: 97.1% (94.5% in males, 97.9% in females) in 2012, 96.1% (92.4% in males, 97.3% in females) in 2012, and 92.4% (84.9% in males, 94.8% in females) in 2010, respectively. Though male thyroid cancer patients showed a relatively poor prognosis compared to female cases, their survival rate improved (5-year survival rate 84.5% in 1997, 84.9% in 2010), with an average APC of 0.66 (0.00-1.32; p<0.05).

**Table 10:** Joinpoint analyses of thyroid cancer survival by calendar year, sex, and Charlson Comorbidity Index (CCI) in Taiwan, 1997-2012

		Thyroid cancer overall survival		Average APC	Trend1		Trend2		Trend3	
		1997	Last year^a^		Years	APC (95%CI)	Years	APC (95%CI)	Years	APC (95%CI)
**Total**	1 year survival rate	94.9% (93.30 to 96.11)	97.1% (96.44 to 97.67)	0.19 (0.08 to 0.31)*	1997-2012	0.19 (0.08 to 0.31)*				
	2 year survival rate	93.7% (92.02 to 95.11)	96.1% (95.38 to 96.79)	0.16 (-0.09 to 0.42)	1997-2004	-0.25 (-0.77 to 0.28)	2004-2012	0.53 (0.24 to 0.81)*		
	5 year survival rate	90.2% (88.14 to 91.92)	92.4% (91.22 to 93.47)	0.20 (-0.18 to 0.57)	1997-2003	-0.45 (-1.21 to 0.32)	2003-2010	0.75 (0.30 to 1.22)*		
**Sex**	**Male**									
	1 year survival rate	90.7% (85.61 to 94.02)	94.5% (92.46 to 96.00)	0.44 (0.17 to 0.71)*	1997-2012	0.44 (0.17 to 0.71)*				
	2 year survival rate	90.2% (85.00 to 93.60)	92.4% (90.05 to 94.16)	0.50 (0.14 to 0.86)*	1997-2012	0.50 (0.14 to 0.86)*				
	5 year survival rate	84.5% (78.53 to 88.86)	84.9% (81.36 to 87.74)	0.66 (0.00 to 1.32)*	1997-2010	0.66 (0.00 to 1.32)*				
	**Female**									
	1 year survival rate	96% (94.29 to 97.14)	97.9% (97.20 to 98.41)	0.14 (0.06 to 0.21)*	1997-2012	0.14 (0.06 to 0.21)*				
	2 year survival rate	94.6% (92.80 to 96.03)	97.3% (96.49 to 97.86)	0.15 (0.02 to 0.28)*	1997-2005	-0.09 (-0.31 to 0.13)	2005-2012	0.42 (0.24 to 0.60)*		
	5 year survival rate	91.6% (89.45 to 93.40)	94.8% (93.61 to 95.76)	0.23 (0.01 to 0.46)*	1997-2006	-0.03 (-0.29 to 0.22)	2006-2010	0.84 (0.24 to 1.45)*		
**CCI**	**CCI ≤ 3**									
	1 year survival rate	96.3% (94.90 to 97.27)	97.9% (97.30 to 98.38)	0.15 (0.05 to 0.25)*	1997-2012	0.15 (0.05 to 0.25)*				
	2 year survival rate	94.6% (93.01 to 95.82)	97.2% (96.48 to 97.73)	0.26 (-0.25 to 0.77)	1997-2000	0.86 (-0.59 to 2.34)	2000-2003	-0.94 (-3.45 to 1.64)	2003-2012	0.46 (0.29 to 0.63)*
	5 year survival rate	91.4% (89.55 to 93.01)	94.3% (93.18 to 95.20)	0.41 (-0.37 to 1.18)	1997-2000	1.16 (-0.84 to 3.20)	2000-2003	-1.28 (-4.74 to 2.31)	2003-2010	0.81 (0.44 to 1.19)*
	**CCI ≥ 4**									
	1 year survival rate	81.4% (72.18 to 87.88)	83.6% (76.92 to 88.56)	0.20 (-0.33 to 0.72)	1997-2012	0.20 (-0.33 to 0.72)				
	2 year survival rate	79.4% (69.89 to 86.17)	78.6% (71.39 to 84.22)	-0.02 (-0.67 to 0.63)	1997-2012	-0.02 (-0.67 to 0.63)				
	5 year survival rate	69.1% (58.84 to 77.25)	66.4% (57.54 to 73.84)	-0.12 (-1.19 to 0.95)	1997-2012	-0.12 (-1.19 to 0.95)				

APC, annual percent change; CI, confidence interval; CCI, Charlson Comorbidity Index. *P<0.05.

^a^ The last year for the 1-year and 2-year survival rate is 2012; the last year for the 5-year survival rate is 2010.

**Figure 8:**
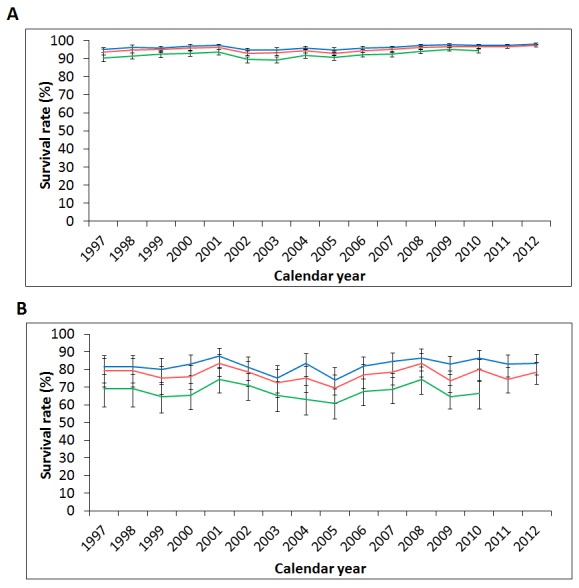
Secular trends of the one-, two-, and five-year survival rates of thyroid cancer in patients with **(A)** Charlson Comorbidity Index (CCI) ≤ 3; **(B)** CCI ≥ 4 in Taiwan from 1997 to 2012. (Blue: 1-year survival rate, red: 2-year survival rate, green: 5-year survival rate.)

In view of the potentially significant impact of comorbidities on overall survival, we next divided our study populations into 2 groups according to their CCI score. As a result, the CCI ≤3 group (88.5% of thyroid cancer patients) showed slightly better survival rates than the overall study population, while the CCI ≥4 group (11.5%) showed much lower survival rates (1-, 2-, and 5-year survival rates: 83.6%, 78.6%, 66.4%, respectively) (Figure 8).

## DISCUSSION

### Interpretation of current results

In our present population-based cohort study combining data from the Taiwan NHI database, the Taiwan Cancer Registry, and the National Death Registry, the reported age-standardized incidence rate of thyroid cancer was 12.30 per 100,000 person-years in 2012, with an average APC of 5.1 (95% confidence interval, 2.5 to 7.8) during 1997-2012, similar to the incidence and APC reported in the Surveillance, Epidemiology, and End Results (SEER) database of the United States (age-standardized incidence rate, 13.98 per 100,000 person-years in 2012; APC, 5.5 during 1992-2012) [9]. Male patients presented at older age at diagnosis and increased more rapidly than female cases, with the female-to-male ratio of age-standardized incidences decreasing from 4.8 in 1997 to 3.3 in 2012. When comparing the demographic data of patients with different histological subtypes, patients with papillary thyroid carcinoma presented at a younger age, had less comorbidities, and had a higher socioeconomic status. The incident percentage of the papillary subtype increased the most during the study period (from 80.6% of the total cases in 1998 to 89.8% in 2012), and the papillary-to-follicular incidence ratio increased from 8.14 in 1997 to 14.13 in 2012, a ratio even higher than that seen in the SEER database (9.88 during 2005-2009) [10]. Thyroid cancer most frequently affected those with high socioeconomic status, such as those with higher income level, urban residence, and professional occupation, and this disparity got even more pronounced in recent years. Regarding the treatment procedures, the numbers of partial thyroidectomies increased the most (average APC, 17.3), which also implied that the numbers of incident cases with small-sized thyroid tumors increased the most during this period. The survival rates of thyroid cancer patients by calendar year, sex, subtype, and CCI were essentially stable; however, patients in the subgroups of male sex, CCI≥ 4, and aggressive histological subtypes showed worse prognoses. These findings were consistent with epidemiological studies conducted in other countries [11, 12]. Combining the following observations in our study: the increasing thyroid cancer incidences but stable survival, predominant increase in the papillary subtype, and marked increase in patients with high socioeconomic status, our results imply that over-detection of subclinical lesions could largely explain the epidemic of thyroid cancer in Taiwan.

### Analysis of socioeconomic status

Socioeconomic status is highly associated with individual’s access to health care such as screening, preventive care, and treatments. If the observed increase in thyroid cancer incidences is due to enhanced detection, patients with high socioeconomic status would be expected to have a greater increase in the incidence of small papillary tumors, which has been observed in several cohort studies using the SEER database and coincident with our results [13–15]. However, for large tumors (> 4.0 cm), a similar steady increase in incidence was also observed both for patients with high and low socioeconomic status in one previous study [13], indicating the existence of a true increase of thyroid cancer. In Canada, where a universal health care system is utilized, socioeconomic disparities of thyroid cancer were also reported [16, 17]. Patients with thyroid cancer among those with low socioeconomic status were not only less frequently diagnosed but also presented with more advanced stage than patients with high socioeconomic status [17].

### Over-detection of subclinical thyroid cancer

According to epidemiologic data from the global cancer registries of World Health Organization, there is an upward trend in the incidences of thyroid cancer (mainly papillary carcinomas) in both sexes and a stable or downward trend in mortality in most areas of the world [18]. The epidemic of thyroid cancer is often attributed to over-detection of small papillary lesions subsequent to improved diagnostic modalities, increased medical surveillances, and accessible “health checkup” services [19]. Generally, the increase of thyroid cancer incidences is faster in the developed high-income countries, with the exceptions of relatively modest increases in Denmark, the United Kingdom and Japan [12]. In the Asia-Pacific regions, the reported thyroid cancer incidences were higher in the high-income countries of Eastern and Western Asia with easier medical accessibilities, while the incidences were apparently low in the developing countries of South-Central Asia, such as India [2]. The epidemiological variations between different countries are usually ascribed to different health systems and screening practices [18]. Among the countries with reported epidemics of thyroid cancer, South Korea has by far the largest increase in the incidence of thyroid cancer. In South Korea, the incidence rate of thyroid cancer diagnosed in 2011 was 15 times higher compared to that observed in 1993, making thyroid cancer the most commonly diagnosed cancer among South Korean women [20, 21]. These increasing diagnoses largely resulted from fee-for-service providers adding thyroid ultrasonography within the frame-work of organized screening programs for other cancers paid for by the South Korea government since 1999 [20]. Since then, there was 6.4-fold increase in thyroid cancer incidence in South Korea between 1999 and 2008, of which 94.4% were small tumors (<20 mm) mainly detected by screening, and 97.1% of the total increase were locoregional tumors, with five-year relative survival rates over 100% [22].

Using tumor size at diagnosis, the estimated proportion of overdiagnosis in women during 2003-2007 may account for 90% of thyroid cancer incidences in South Korea; more than 70% in the United States, France, Italy, and Australia; and for 50% in Japan [21, 23]. The proportions attributable to increased detection are higher in countries with larger incidence increases and are consistent across both sexes, although the reported increases were often smaller and delayed in men. In another study, the cancer stage at diagnosis was used to evaluate the overdiagnosis of papillary thyroid carcinoma in the SEER database [6]; as a result, the reported proportions of overdiagnosis in 2011 were 5.5% and 45.5% in men aged 20–49 and >50 years, and 41.1% and 60.1% in women aged 20-49 and >50 years, respectively. Overdiagnosis, established by the surplus of early stage diseases outpacing late stage diseases, has resulted in an additional 82,000 incident papillary thyroid cancers during 1981-2011 in the US, and the majority of over-diagnosed patients will not benefit from surgical or adjuvant interventions due to their inherently favorable prognosis [6]. In our study, estimating the magnitude of overdiagnosis was impossible, since the databases used contained no information on the tumor size or cancer stage at diagnosis.

### True thyroid cancer increase: possible causative risk factors

The proposed risk factors of thyroid cancer in the literatures include females, family history of thyroid cancer, radiation exposure, excess weight, iodine insufficiency and dietary factors [12]. However, available evidences on the risk factors of thyroid cancer were limited and heterogeneous. Tobacco use and alcohol consumption were associated with a slightly reduced risk of thyroid cancer in several cohort studies [24, 25]. Diabetes and excess weight have been recognized as risk factors for increased the incidence and mortality of thyroid cancer [26]. In a pooled analysis of 22 prospective cohort studies, greater body height and excess weight throughout adulthood were found to be associated with higher incidences of thyroid cancer (except the medullary subtype), and higher thyroid cancer mortality [27]. Since the incidence of most malignancies did not increase during the same period, the true carcinogens behind the epidemics of thyroid cancer must be thyroid-specific rather than common causes of other cancers such as diabetes, obesity, or viruses. A true increase of thyroid cancer incidence in recent decades, driven by the emergence of potential thyroid-specific risk factors such as radiation exposure, iodine insufficiency, or environmental carcinogens, is supported by molecular and epidemiological evidences.

The molecular evidence comes from a histological study analyzing genetic variations of papillary thyroid carcinomas in a United States institution from 1974 to 2009, which showed sharply rising percentages of the follicular variant histology (from 18% to 44%) and RAS mutations (from 3% to 25%), overall stable prevalence of BRAF mutations but increased percentage within the classic papillary type (from 50% to 77%), and a decreased proportion of RET/PTC rearrangements (11% to 2%) [28]. These results not only suggest the existence of new and more recent etiologic factors resulting in the change of molecular profiling and the increased incidence of thyroid cancer, but also imply that environmental or therapeutic radiation is not likely to be the major contributor, since as many as 80% of papillary thyroid carcinoma patients with radiation exposure carry RET/PTC chromosomal rearrangements [3, 29].

On the other hand, epidemiological observations suggesting a true increase of thyroid cancer incidences include the following: flat mortality rates of thyroid cancer despite earlier diagnosis and more effective therapies [1], similar steady increase in incidence of large thyroid tumors (> 4.0 cm) both in patients with high and low socioeconomic status [14], and the sharp increase in the incidence of papillary thyroid carcinomas 4-5 years after the nuclear-reactor accident in Chernobyl in 1986 [30].

Among these risks factors of thyroid cancer, exposure to ionizing radiation appears to be the most important contributor for papillary thyroid carcinoma with strong evidences support. The thyroid gland is highly sensitive to the carcinogenic effects of nuclear radiation, including diagnostic radiography, nuclear medicine, radiation therapy, and cosmic radiation during flights. After the Fukushima power plant accident in 2011, intensive ultrasonography screening programs within 4 years revealed that the thyroid-cancer incidence among screened adolescents (≤18 years) was approximately 30 times as high as the national average [31]. The nuclear radiation exposure dosage and age at exposure are key factors for the risk of radiation-induced thyroid cancer, and continuous monitoring accompanied with strict regulations of radiation exposure should be emphasized to prevent any health hazards. In Taiwan, a recent study comparing the incidence rates of leukemia and thyroid, lung, and breast cancers between patients in the “plant-vicinity” and “non-plant-vicinity” found no significant differences [32]. These results are consistent with our finding that there was no significant association between the geographic variation of thyroid cancer prevalence and the sites of nuclear power plants in Taiwan. For most people living far from the nuclear reactor/disasters, medical radiations make up a significant proportion of exposure, and its association with thyroid cancer has been exemplified in several studies that healthcare professionals had increased risks of thyroid cancer [33]. Besides, the use of radioactive iodine was associated with higher risk of secondary malignancy among thyroid cancer survivors [34].

Chronic iodine deficiency is firmly established as a risk factor for goiter and follicular thyroid carcinoma [3]. There might be a shift from the follicular to the papillary subtype after decades of universal prophylactic iodization programs [3]. In Taiwan, iodine sufficiency has been maintained since the mandatory salt iodization program implemented in 1967 and provides an essential element of thyroid hormones [8]. However, the iodization policy changed from mandatory to voluntary since 2003 in Taiwan, and the iodine status of Taiwanese populations has become mild insufficiency according to an adult urine iodine survey during 2005-2008 in all geographic areas (except the Southern area and Penghu islands), with significant insufficiency in subgroups of older age and mountain areas [35]. However, iodine deficiency may not be the true cause behind the epidemic of thyroid cancer in Taiwan, since follicular carcinoma was not the predominant increased subtype in recent years.

Therefore, the recent emergence of environmental carcinogens is the most possible candidate risk factors accounting for the epidemic of thyroid cancer in Taiwan. The westernized post-industrial lifestyle has profoundly affected the dietary habits in Taiwan, and the increased nitrite and nitrate ingestion through drinking water contaminated by fertilizers and through processed food has already been associated with an increased risk of thyroid cancer [36]. Besides, residents in volcanic areas are at higher risk of papillary thyroid cancers, suggesting that a volcanic environment with trace elements or heavy metals may play a role in the carcinogenesis of thyroid cancer [37]. These potential thyroid-specific carcinogens, such as solvents, pesticides, plastic constituents, and heavy metals, might interfere with our hormone homeostasis and increase the thyroid susceptibility to environmental carcinogens. For example, Polybrominated diphenyl ethers, a fiame retardant commonly used in a variety of textiles and household products, are recognized to alter thyroid hormone homeostasis, but its association with thyroid cancer was not proved in a large-scale case-control trial [38]. Therefore, so far, no solid evidence for thyroid-specific carcinogens or carcinogenesis has been identified.

### Treatment strategies for patients with thyroid cancer

According to the American Joint Committee on Cancer TNM staging for thyroid cancer (7th edition, 2010), thyroid cancers are staged based on the primary tumor size and presence of extra-thyroid extension. For T0 patients (no evidence of primary malignant tumor), no local surgery or radiation treatment is recommended. Thyroid lobectomy alone is recommended for patients with thyroid microcarcinoma, but surgeons often perform total thyroidectomy in most clinical settings (74.3%), and more than one-fifth of patients reportedly received additional radioiodine ablation in the SEER database [39, 40]. However, thyroid cancer-specific survival analysis showed no survival advantage in those patients with thyroid microcarcinoma receiving surgery or radiation therapy [40]. Lobectomy or total thyroidectomy is the treatment of choice for primary thyroid cancers that measure 1-4 cm in the greatest dimension; however, aggressive total thyroidectomy is preferred, because it can reduce the risks of local recurrence and nodal metastasis. Total thyroidectomy with neck dissection is recommended for tumors larger than 4 cm in the greatest dimension or for cases of evident locoregional metastasis. Thyroidectomy carries a 1-6% risks of complications, including hypothyroidism, hypoparathyroidism, and vocal cord paralysis, while lobectomy is associated with complications in roughly half as many risks [41]. The 2015 American Thyroid Association (ATA) management guidelines recommend that thyroid lobectomy alone may be sufficient as the initial treatment for low-risk, unifocal, and intrathyroidal differentiated thyroid carcinomas <4 cm, unless there are clear indications to remove the contralateral lobe [42].

Iodine-131 is a unique radioactive iodine that targets thyroid tissue and is widely used to eliminate occult residual tumors in order to reduce the risk of recurrence. Radioactive iodine is also used to treat persistent diseases and to ablate any remnant thyroid tissue, thereby facilitating further surveillance by serum thyroglobulin or radioiodine whole-body scintigraphy [3]. Postoperative adjuvant radioiodine ablation is recommended for T4 or metastatic patients, but is not recommended for tumors < 1cm, and is not routinely recommended for tumor < 4 cm in diameter after lobectomy or total thyroidectomy in the absence of other adverse features [42]. For low-risk thyroid cancer, recombinant human thyrotropin and postoperative low-dose (30mCi) radioiodine ablation may be sufficient for ablating the remnant thyroid [43]. BRAF-mutated cancers and those that are positive on fluorodeoxyglucose positron emission tomography scans are often refractory to radioiodine [3].

Patients with a low to intermediate risk disease are monitored by neck ultrasonography and assessment of their serum thyroglobulin levels [3]. Thyroid cancers are often indolent, even when they have metastasized to distant sites. Currently, systemic therapy is reserved for patients with metastatic disease that is progressing, symptomatic, or threatening vital structures and that is not amenable to localized therapies. Palliative radiotherapy alone, combined radiotherapy and low-dose chemotherapy, or local therapies may control the disease in patients with unresectable regional or metastatic disease. With the advances in molecular genetics, molecular targeted therapy with tyrosine kinase inhibitors such as sorafenib, lenvatinib, vandetanib, and cabozantinib has been introduced for radioiodine-refractory patients to control tumor progression and prolong disease-free survival [3]. In the future, the results of genetic profiling will be incorporated into this stratified process [44]. For example, a mutation in the telomerase reverse transcriptase (TERT) promoter is an independent indicator of poor prognosis for all differentiated thyroid carcinomas [45].

In 2006, the ATA first published their management guidelines for patients with thyroid nodules and differentiated thyroid cancer [46]. In these guidelines, total or near-total thyroidectomy was recommended as the initial surgical procedure for most thyroid cancer patients, and thyroid lobectomy alone was recommended for small (<1 cm), low-risk, intrathyroidal tumors in the absence of nodal metastases; routine central neck dissection was suggested for patients with papillary thyroid carcinoma; and radioiodine was recommended for patients with stage II-IV disease. The release of the 2006 ATA guidelines changed the clinical management for differentiated thyroid cancer in the US, with significantly more patients receiving total thyroidectomy, lymphadenectomy, and radioiodine after 2006, as disclosed by analysis of the SEER database [47]. In our study, the joinpoint analysis of thyroid cancer treatments showed accelerating trends of total thyroidectomy, neck dissection, and radioiodine ablation accompanied by a decelerating trend of partial thyroidectomy after 2006; these findings may be coincident or be related to the publication of the 2006 ATA guidelines. It is possible that the trends in thyroid cancer management in Taiwan might change again after the 2015 ATA guideline revision, with less use of extensive surgeries and radioiodine ablation, but more utilization of tyrosine kinase inhibitors [48].

### Prognostic factors for patients with thyroid cancer

In our analyses, the prognostic factors for overall survival of thyroid cancer patients were identified as the histological subtype, age at diagnosis, sex, and comorbidities. Among these, the histological subtype was the most significant prognostic factor. In a multivariate analysis of the SEER database during 1988-2007, the reported independent prognostic factors for thyroid cancer-specific survival were as follows: age>45 years, aggressive histopathology, advanced tumor stage, tumor size > 4 cm, extrathyroidal extension, lymph node or distant metastasis, and postoperative radiation [49]. Advanced age is associated with poor prognosis in patients with papillary thyroid carcinoma, and a transcriptional profiling study showed that metabolic alterations and immune dysregulation are possible underlying mechanisms [50]. Among these variables to stratify the risks of recurrence, several molecular markers have shown promise. Especially, mutations of the TERT promoter, RET, and BRAF are established as major prognostic biomarkers and are associated with clinical aggressiveness of papillary thyroid carcinoma [51, 52]. In Chinese patients with papillary carcinoma, TERT promoter mutations rather than BRAF V600E mutations were associated with a more advanced TNM stage and shorter progression-free survival [53].

### Future aspects for thyroid cancer managements

The increase in health care expenditures related to over-detection and management of these presumably low-risk thyroid cancers with possibly unfavorable treatment outcomes has resulted in a backlash trend against the over-detection and over-treatment for thyroid cancer. Reports from Japan comparing active surveillance with immediate surgery for more than 2,000 patients with low-risk papillary microcarcinoma showed equally excellent oncological outcomes and fewer unfavorable events [54]. However, before being confirmed by a large-scale prospective study, the active surveillance approach should be considered the preferred option for patients with low-risk papillary thyroid carcinomas. Diagnostic risk-stratification can help identify the patients who should be considered for aggressive management or watchful waiting. Future investigations should include evaluations of thyroid cancer molecular prognostication and identification of novel thyroid-specific carcinogens, randomized clinical trials for evaluating the outcome of active surveillance approaches, and studies aimed at identifying better diagnostic criteria to recognize and differentiate subclinical thyroid lesions from progressing and invasive cancers. With time, molecular profiling of fine-needle aspiration specimens or postoperative samples for patients with thyroid cancer will facilitate the development of risk-stratified management and individualized treatment strategies.

### Limitations of this study

There are several limitations to our study. First, the NHI research database contains no clinical information on cancer stage, tumor size and histological subtype, and information about cancer stage and tumor size of thyroid cancer is still unobtainable from the Taiwan Cancer Registry database, not to mention that molecular profiling of thyroid cancer is still not generally performed for thyroid cancer specimens in Taiwan. Therefore, we could not evaluate the associations of thyroid cancer stage and tumor size with their corresponding survival rates, or estimate the magnitude of overdiagnosis by determining the tumor size or stage at diagnosis. However, the other presented available clinical information of thyroid cancer from the NHI database has excellent agreement with those from the Taiwan Cancer Registry. Those data are inherently complementary and are supported by consistent epidemiologic statistics between previous studies and our results.

Second, the treatment procedures for thyroid cancer presented herein included those administered after the diagnosis of thyroid cancer. However, treatments might take place before the diagnosis of thyroid cancer in clinical practice, such as thyroid operations for benign diseases. Therefore, the presented numbers of treatment procedures for thyroid cancer likely underestimate the true situation, especially in terms of the numbers of thyroid lobectomies and total thyroidectomies. Additionally, because of the lack of environmental pollutions and radiation exposure data, we were not able to assess the associations between environmental carcinogens and the incidence of thyroid cancer. Besides, there is a potential for coding errors in the analysis of large national databases, which might possibly result in underestimation of the incidence and treatment procedures of thyroid cancer, even though these nationwide datasets are reported with standardized abstraction methods and are highly scrutinized. Finally, since we obtained information of thyroid cancer patients from various databases, the creation of a risk stratification models of prognostic factors for thyroid cancer-specific survival became impossible, because the eligible populations in these databases differed.

Despite these limitations, this is, to our knowledge, the first large-scale epidemiological study of thyroid cancer in Taiwan combining nationwide data from the NHI database, the Taiwan Cancer Registry, and the National Death Registry, making the reported findings more generalizable for the Taiwanese population. To eliminate the risk of underestimation of the survival rates from the NHI database, we crosslinked the National Death Registry to obtain the most accurate mortality rates. We also provided abundant clinicodemographic information, including information on the patients’ socioeconomic status, histological subtypes, comorbidities, and geographic variations, to make our present report more comprehensive and trustworthy.

## CONCLUSION

In this epidemiological study of thyroid cancer in Taiwan during 1997-2012, we found 2.2- and 4.2-fold increases in the incidence and prevalence of thyroid cancer, respectively, especially predominant in the papillary subtype. The female-to-male ratio of age-standardized incidence rates decreased from 4.8 in 1997 to 3.3 in 2012. The male patients presented at older age at diagnosis and had worse prognosis than female cases. Thyroid cancer was more prevalent in patients with high socioeconomic status and this disparity grew even more pronounced in recent years. The overall survival rates by sex and subtype remained stable over time, with 5-year survival rates of 90.2% in 1997 and 92.4% in 2010. Taken together, the rising incidence (predominant in the papillary subtype) but stable survival rates, and the marked increase in high socioeconomic status, imply that enhanced detection of subclinical lesions is the major contributor for the epidemic of thyroid cancer in Taiwan, possibly due to improved diagnostic modalities and increasing medical surveillance. A true increase of thyroid cancer due to thyroid-specific environmental carcinogens might also play some role, but warrant further investigations.

## MATERIALS AND METHODS

### Ethics statement

This epidemiologic study was approved by the institutional review board of Chang Gung Memorial Hospital (approval number: 201601563B1). As the data used in this study were anonymized, the need for patient consent was waived.

### Data sources and study population

Thyroid cancer cases were identified from the Taiwan NHI Research Database between 1997 and 2012. This database routinely collects health information from all Taiwan NHI beneficiaries since 1995. The NHI is a single medical expense payer system that covered over 99.6% of beneficiaries registered in the database at the end of 2014. The NHI research database includes demographic data and information regarding the diagnoses, operations, and prescriptions from both primary and specialist care providers. The personal information in the NHI database during the study period was encrypted and encoded using the International Classification of Diseases, Ninth Revision, Clinical Modification (ICD-9-CM). The validity, representativeness, and clinical consistency of this database have been reported in many previous studies. We also crosslinked the Taiwan Cancer Registry database to gain information about the histological subtypes of thyroid cancer. The survival rates of thyroid cancer patients were obtained from the National Death Registry database.

### Case definition of thyroid cancer

The thyroid cancer cases were identified by using the ICD-9-CM code 193 in the Catastrophic Illness Registry of the NHI database between 1997 and 2012. The Catastrophic Illness Registry is a medical co-payment waiver provided by the Taiwan government in order to reduce the economic burden of patients with severe diseases, including thyroid cancer. Medical professionals should provide pathological or cytological reports to expert panels before approval of waivers. The associated clinical information including the unique personal identification, diagnosis, demographics, application date, diagnosing physician, hospital, and other administration data, is sent to the insurance administration.

### Estimation of the prevalence, incidence, and survival rate

The crude prevalence rate of thyroid cancer per 100,000 individuals was calculated by using the number of prevalent cases divided by the eligible population in that specified calendar year. We defined thyroid cancer patients as individuals who had a medical record of thyroid cancer before July 1 of each calendar year. The denominator for the prevalence estimation was defined as all individuals registered on July 1 of each calendar year. The crude incidence rate of thyroid cancer per 100,000 person-years was calculated by using the number of incident cases divided by the total person-years in an at-risk population accumulated during that same year. Patients with no diagnosis of thyroid cancer prior to January 1 of each calendar year and with a record of thyroid cancer during that year were defined as incident cases. People with no history of thyroid cancer for each calendar year during the same year were defined as our at-risk cohorts. The follow-up was calculated from January 1 of the year of the earliest date of thyroid cancer diagnosis until death or December 31 of that specified year. To diminish the restrictions of the databases, we only included patients with at least a one-year registration period prior to January 1 of each calendar year in our eligible cohorts. The age-standardized prevalence and incidence of thyroid cancer were calculated in each calendar year between 1997 and 2012, with the population structure in 2012 as the reference. Data about histological subtypes were obtained from the Taiwan Cancer Registry database. Subgroup analyses of prevalence and incidence were conducted according to sex, calendar years, and histological subtypes. The mortality rates of thyroid cancer patients were obtained from the National Death Registry. We estimated the one-year, two-year, and five-year survival rates in the different calendar years using the life table method. We also calculated the geographic variations in prevalence and incidence of thyroid cancer in 1997 and in 2012 by dividing Taiwan into 21 cities and counties, including Taipei city, New Taipei City, Keelung city, Taoyuan county, Hsinchu city and county, Miaoli county, Taichung city, Taichung county, Changhua county, Yunlin county, Nantou county, Chiayi city and county, Tainan county, Tainan city, Kaohsiung county, Kaohsiung city, Pingtung county, Yilan county, Hualien county, Taitung county, and offshore islets (Penghu). To eliminate the effects of different age and sex structures in various regions, we estimated the age-standardized prevalence and incidence of thyroid cancer with respect to the overall population structure of 2012.

### Estimation of treatment procedures for thyroid cancer

We defined five different treatment procedures for thyroid cancer using the ICD-9-CM diagnosis or procedure codes, including partial thyroidectomy (procedure codes: 062, 063), total thyroidectomy (procedure code: 064), lymph node dissection (procedure codes: 403, 404, 405), chemotherapy (diagnosis codes: V581, V073), and external beam radiotherapy (diagnosis codes: V580, V661, V671). We used the codes of the NHI payment system to define patients receiving I-131 ablation therapy. Subsequently, the number of different treatment procedures performed after the diagnosis of thyroid cancer in that specific calendar year were calculated and the average APCs of the treatments were compared.

### Statistical analysis

We estimated the 95% confidence intervals for the prevalence and incidence of thyroid cancer under the assumption of a Poisson distribution. The Joinpoint Regression Analysis program (version 4.0.4) was used to estimate trends for the prevalence, incidence, survival rates, and the number of treatments of thyroid cancer. This program utilizes the Bayesian Information Criterion to generate different numbers of ‘joinpoints’ when the linear trend of the prevalence, incidence, survival rate, or number of treatments of thyroid cancer changes significantly and calculates the average APC for each segment [55]. The secular trends for survival rates were calculated for patients with a CCI score of 0–3 vs. ≥ 4. The CCI score, composed of 17 diagnostic criteria, is often used to evaluate patients’ medical burden and to estimate the risk of death. The statistical significance level was set at 0.05. All statistical analyses were conducted using SAS statistical software (version 9.4; SAS Institute, Cary, NC, USA).

## Abbreviations

(NHI): National Health Insurance
(APC): annual percentage change
(CCI): Charlson Comorbidity Index
(ICD-9-CM): International Classification of Diseases, Ninth Revision, Clinical Modification
(SEER): Surveillance, Epidemiology, and End Results
(ATA): American Thyroid Association
(TERT): telomerase reverse transcriptase
